# Advanced Technologies for Stabilization and High Performance of Seawater RO Membrane Desalination Plants

**DOI:** 10.3390/membranes11020138

**Published:** 2021-02-16

**Authors:** Hiroo Takabatake, Masahide Taniguchi, Masaru Kurihara

**Affiliations:** Toray Industries, Inc. Otsu, Shiga 520-0842, Japan; masahide.taniguchi.x9@mail.toray (M.T.); masaru.kurihara.z9@mail.toray (M.K.)

**Keywords:** seawater desalination, reverse osmosis membrane, seawater intake, pre-treatment, RO process

## Abstract

Seawater desalination plants that use reverse osmosis (RO) membranes have become a core part of social infrastructure, and should be designed to meet the needs of product water quality and production capacity, while considering various environmental factors such as the seawater quality, temperature and geographical features. Furthermore, stable operation while overcoming various problems should be achieved alongside the increasing demands for energy saving and cost reduction. As no universal plant apparatus and operation technology meets these various requirements, the plants need to be customized for individual solutions. This paper reviews and summarizes the proven technologies, including their advantages/disadvantages, and points to cutting-edge technologies related to the design and operation maintenance of seawater intake, pre-treatment and the RO desalination process.

## 1. Introduction

The RO membrane was invented in 1960 by S. Loeb and S. Sourirajan and has been used for the purpose of the seawater desalination [1]. One of the most symbolic events in the history of seawater desalination was the famous speech delivered by the US President J.F. Kennedy in 1961 to approve seawater desalination as a national project. It was a human dream to produce drinking water from seawater at that time. This dream has now come true and seawater desalination is an essential part of social infrastructure throughout the world.

**Figure 1:**
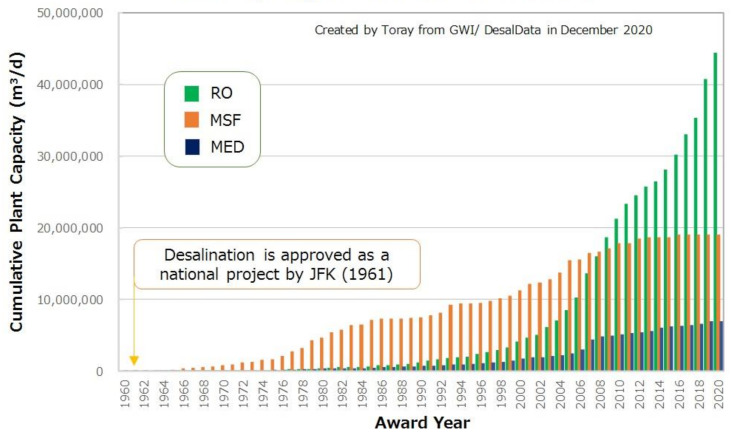
The trend in plant capacity for seawater desalination, created by Toray from GWI/DesalData in December 2020 [2,3].

Seawater desalination in the 1960s was based on distillation technologies, using heat to produce freshwater. The most proven one is Multi Stage Flash Distillation (MSF), which distills seawater by flushing seawater into steam in multiple stages of a heat exchanger. A more effective way is Multiple-Effect Distillation (MED). This consists of multiple stages and distills seawater by heating it in a tube at each stage. The vapor is reused in the next stage to enhance energy efficiency. MED has many advantages, such as higher thermodynamic efficiency and lower temperature, allowing use of low cost materials and heat energy. Such distillation methods were the main technologies used for seawater desalination in the world until the 2000s (see Figure 1 [2,3]).

Seawater desalination plants using RO membranes (hereinafter called seawater RO (SWRO) membrane) appeared around the 1970s. For freshwater production from seawater via a SWRO membrane, the sum of the osmotic pressure of seawater and the pressure for membrane permeation needs to be applied to seawater. Such pressure is very high (currently 5–7 MPa), and significantly impacts the energy consumption and operating costs of the desalination plant.

Until the 1980s, desalination plants using SWRO membranes were very limited because the initial/operating costs and energy consumption of the SWRO membrane desalination were not cost competitive compared to the thermal desalination process, mainly due to the high price and insufficient performance of the SWRO membrane. However, SWRO membrane desalination has exponentially expanded since the 1990s, as shown in Figure 1, due to improvement in the SWRO membrane performance, leading to a reduction in energy consumption, as well as a decreased production cost of SWRO membranes. In addition, innovative energy saving technology with energy recovery devices (ERD) assisted the expansion of SWRO desalination. The continuous innovation and improvement of SWRO membranes and plant technologies has brought about a steady expansion of SWRO desalination, and almost all new desalination plants, in particular, large plants with more than 100,000 m^3^/day of production capacity, since 2000 were SWRO membrane desalination plants [3,4,5]. The current production capacity of SWRO seawater desalination plants has exceeded 40 million m^3^/day in the world, and is still growing significantly as shown in Figure 1 [2,3].

Water treatment plants including SWRO desalination plants need to be customized to respond to users’ demands, such as product water quality, energy saving, cost reduction, and stable operation under a variety of environmental conditions and seawater qualities. Therefore, it is difficult to establish a universal design or standard operation philosophy for SWRO desalination plants. As not only the RO process itself, but also the seawater intake and pre-treatment are very important for stabilizing the RO process operation, this review covers the technologies that contribute to RO process stabilization, energy saving, and cost reduction, mainly focusing on the basic system configuration, their features, and recent tendencies.

## 2. Basic History of SWRO Membrane Development

As the core technology of SWRO membrane desalination plants is, of course, the SWRO membrane, the knowledge of the basic history of SWRO membranes is essential for understanding SWRO plant technologies. Thus, a timeline is given in Table 1.

Exploratory research on RO membranes started in the 1950s at the University of Florida, followed by Sydney Loeb and Srinivasa Sourirajan at the University of California, Los Angeles (UCLA), in the United States, and an initial type of cellulose acetate (CA) asymmetric membrane was developed in 1960 [1]. In the commercial sector, General Atomics (acquired by UOP, now Koch) began the commercialization of CA flat sheet membranes (spiral-wound type) in the middle 1960s. At almost the same time, DuPont commenced research on RO membranes and developed linear polyamide (PA) hollow fibre membranes. They launched this membrane in 1968 [1], but then stopped the SWRO membrane business later. Later in the 1960s, Toray began research and development on a spiral-wound type element of CA and PA flat sheet membranes. The RO membrane development at Nitto Denko Corporation and Toyobo Co., Ltd. began in 1973 and 1972, respectively [1].

In the 1970s to the 1980s, research and development on the composite membrane accelerated and the membrane performance was enhanced. In 1977, Cadotte, who later established FilmTec Corporation (which was acquired by Dow, and then DuPont), and his colleagues succeeded in developing a cross-linked aromatic polyamide (PA) membrane [1,6], which is the basic structure of today’s membranes. On the other hand, Toray developed a cross-linked polyether composite membrane, named “PEC-1000”, with superior performance in salt rejection and water permeability [1]. However, this membrane was very sensitive to dissolved oxygen and required very careful attention when handled in seawater desalination plants [1,7,8]. In order to overcome such a drawback, Toray created ultra-thin cross-linked aromatic polyamide (PA) composite membranes by in-situ interfacial polymerization with 1,3,5-triaminobenzene as polyamine and polyfunctional acid chlorides [1,8,9]. This membrane had outstanding performance as well as sufficient durability [8,9], and formed the basis of Toray’s current SWRO membrane.

On the other hand, technical advances were made on hollow-fibre type membranes made of cellulose tri-acetate (CTA) with superior oxidation tolerance and a greater membrane area in the element [1]. CTA hollow fibre membranes were mainly used in seawater desalination plants in Saudi Arabia, however they required frequent chemical cleaning due to the poor seawater quality with a high concentration of organic matter. Therefore, PA flat sheet membranes with a spiral type configuration became the main membrane used in the rest of the world because they have high separation potential with low price per productivity. Products of the said membranes specialized in energy saving (high water permeability) and high product quality, and they resulted in a cost reduction for plant operation [1]. In this field, Toray, DuPont (formerly Dow), Hydranautics (Nitto Group Company), and LG (acquired Nano H_2_O) currently occupy more than 90% share in manufacturing and supplying PA composite membranes (spiral wound type) [10], and the competition among these companies has become fierce.

## 3. Typical Types of Seawater Intake and Their Advantages and Notices

The seawater intake process supplies seawater with stable quantity and quality to desalination plants, and it plays important roles in stabilizing the entire desalination system. It is important to take into consideration various factors such as the geography, seawater characteristics, tidal currents, and impacts on the marine ecosystem when designing a suitable intake system. The most common seawater intake system uses surface seawater because of its convenience. The seawater intake is preferably set at a depth of more than 10 m and at approximately 5 m above the seabed in order to avoid sand and sludge from the seabed getting sucked in, even when tidal currents change [11]. It is usual to locate the intake point at a distance of 0.1–1 km from the shore to suppress the impact of such disturbances and pollution. Setting the intake point further from the coast incurs higher capital cost. If the coast is shallow, the cost benefit should be analyzed to determine the optimum distance [11].

The capital cost of surface seawater intake is relatively low but tidal currents and seasonal changes may cause large fluctuations in the seawater quality. Depending on the location, the tidal zone and prevailing wind direction might also affect the seawater quality and lead to “fouling” (deterioration in the membrane performance) in the RO process. Furthermore, the environmental impact of seawater intake and concentrated discharge water (brine) from desalination plants should be assessed carefully [11,12].

Other methods of avoiding such problems include “deep seawater intake” and “beach well intake.” Deep seawater (more than 200 m deep) is relatively stable, lower in temperature, and better quality, without being affected by the climate and discharge from the land, which may lead to less RO fouling [13]. Beach well intake involves drawing up seawater from below the level of the seabed. Such seawater has already passed through the seabed, which acts as a slow sand filter, as described later, and blocks the intrusion of marine creatures. However, for both deep seawater intake and beach well intake, the capital cost is high and the local geography limits areas where they can be established. Furthermore, in the case of a beach well, the surrounding seabed gradually becomes clogged and another new well must be drilled to secure the intake water capacity [14]. Therefore, comprehensive environmental and economic assessments are essential when installing beach well intakes. For example, Shahabi et al. [15] provided a comparative life cycle assessment between the scenarios of open intake and beach well intake for a 35,000 m^3^/day capacity desalination plant, and they concluded that the levelised cost of the beach well intake was 13% lower than that of the open sea intake. Missimer et.al. [16] reported that the beach well intake system gives a 5–30% reduction in operating cost and decreases the environmental impact of discharged brine water due to a smaller amount of chemical dosage.

Maintaining the seawater intake pipeline to the pre-treatment process is also important for stabilizing the RO process. Fishes intrude into the pipeline, seashells and microorganisms become attached to and grow in the pipeline, and their metabolites may pollute (foul) the RO process. In order to suppress such pollution, a large fish catching apparatus and a screen to block the intrusion of marine creatures should be set. At the same time, chlorination and periodical pipeline cleaning are necessary to suppress the growth of seashells and microorganisms in the pipeline.

The application of continuous chlorination was most often used in the 1990s, but it was reported that chlorination induced the growth of chlorine-tolerant bacteria and produced substrates for microbial growth. Therefore, it may end up promoting biofouling (performance deterioration due to microorganisms and their secretions) in the RO process [13]. At present, the use of chlorination is minimized by intermittent chlorine dosing [17] and the starvation method [18]. In addition, it has been reported that no chlorine dosing is the best way to suppress biofouling [5,13,19]. An adequate chemical dosage is required to reduce chemical costs and the environmental impact. For example, at Dhekelia Seawater Desalination Plant in Cyprus, chlorination was applied for 8 to 24 h every other day at the start of the operation, which caused RO fouling, but it was improved after chlorination was reduced to 4 h twice per month [19,20]. Where geographically possible, it is preferable to retain seawater for 1 day or more in a seawater reservoir or lagoon so that sediment settles and microorganisms and organic matter degrade. This helps to reduce the environmental impact and ensure a stable seawater intake [11].

## 4. Pre-Treatment Process

### 4.1. Typical Pre-Treatment Processes

The main role of pre-treatment is to remove RO foulants, which are the substances such as suspended solids and organic matters that cause RO membrane fouling. Thus, the pre-treatment process is very important to achieve stable operation of the subsequent RO process. Typical pre-treatment processes are sand filtration, membrane filtration, coagulation and sedimentation, dissolved air floatation (DAF), and their combination (as shown in Table 2). Pre-treated seawater is normally supplied to the RO process (described later in detail) via a safety cartridge filter (SF), which consists of 1–20 micron filters to protect high pressure pumps and RO membranes from damage by fragments that leak through the upper flow.

The most proven method is sand filtration, where multi-media filters (MMF) using a combination of various filtration media of different sizes and densities are commonly used [11]. Many plants apply dual media filters (DMF), which are usually 1 mm diameter anthracite (rarely pumice) and 0.5 mm diameter silicate sand stacked on gravel. Smaller sized heavy media such as garnet can be added as the third layer in order to further improve water quality, but this is not common due to the high cost. In some cases, single sand filtration with sand less than 0.5 mm in diameter (polishing filter: PF) is also used following DMF. An important feature of sand filtration is that it is necessary to adopt a combination of coagulation and flocculation because the filtration accuracy is the gap between the sub-millimetre size of the filter media and unstable treated water quality, especially after cleaning the filter media, and the coagulation and fluctuation efficiency change according to the quality of the seawater.

More plants are adopting membrane filtration processes such as micro-filtration (MF) and ultra-filtration (UF) instead of or in addition to sand filtration. Because membrane filtration processes (smaller than 0.1 micron) are finer than sand filtration, the membrane filtration is able to remove suspended solids without coagulation and the treated water quality is relatively stable. However, coagulant is also dosed in most cases even for membrane filtration processes as well as sand filtration to remove dissolved organic matter and reduce RO foulants [11]. Ferric chloride is widely used as an economical coagulant, and the pH of the seawater is sometimes adjusted to improve the coagulant efficiency to around 6.0 to 6.5 [11]. Polymer type coagulants and/or coagulant aids are also useful, but some types of polymer coagulants/aids might adsorb onto the RO membrane surface and deteriorate the RO membrane performance. In both sand filtration and membrane filtration, most coagulants are not effective enough on neutral charge components, and the coagulant dosage increases the chemical cost as well as the environmental impact. Therefore, adequate dosage of coagulants is important for stable and economic plant operation.

DAF and sedimentation processes are adopted to separate the flocs after coagulation and flocculation with different densities, and are commonly equipped primarily to implement sand filtration or membrane filtration. DAF removes coagulated flocs floating on the water surface as they attach to fine air bubbles by hydrophobicity. DAF is widely applied to remove high concentrations of organic matter in wastewater treatment processes, and some of the large size seawater desalination plants install DAF to stabilize the plant operation even during the vigorous growth of microorganisms in the sea, red tides, and oil pollution. Installing DAF incurs additional capital costs and makes operational costs higher, but it enables one to obtain high quality treated water on a stable basis. As described above, all pre-treatment processes of seawater desalination have advantages and disadvantages; therefore, it is necessary to select the optimal process in accordance with the requirements and situation (see Table 2).

### 4.2. Water Quality Indices of the Pre-Treatment Performance

#### 4.2.1. Silt Density Index (SDI)

Water quality indices are very important for judging whether the pre-treatment operation is good or not. However, there is no universal and perfect index at this point due to the high complexity of the RO fouling. The RO fouling is basically classified into colloidal (particulate) fouling, scaling, organic (chemical) fouling, and biofouling [28]. Many studies have been conducted on adequate indices [28], however, only descriptions of typical and basic indices are available.

The silt density index (*SDI*) is the most widely used to judge whether pre-treated water is suitable for RO feedwater. *SDI* is an indicator of overall content in the water, mainly colloidal matter, where particulate accumulation onto membrane surface deteriorates membrane performance. It was defined at ASTM4189-95 [29] as a water quality index. *SDI* is used to measure the decline in the filtration rate over a certain period of time (*s* minutes: 15 min in general) while sample water is filtered, and is calculated by Equation (1):

(1) $$SDI_{S}=\frac{100\times \left(1-\frac{t_{i}}{t_{s}}\right)}{s}$$

where *t_i_*, *t_s_* [*s*] are the time to filter 500 mL of water at the initial time or after *s* minutes.

*SDI* is commonly used as a water index in the SWRO business to indicate the goal of pre-treatment performance and for warranty. However, *SDI*, defined at ASTM4189-95, includes vague parts, and has been modified as a standard method. For example, the filter type to be used was determined at ASTM 4189-07 [30]; however, *SDI* values do not completely indicate the tendency for RO fouling in the actual setting. For example, in Dhekelia Seawater Desalination Plant in Cyprus, fouling occurred even though the *SDI* values were low (less than 2.0). At that time, changing coagulants from ferric chloride to polymer reduced the fouling and achieved stable operation, although *SDI* increased to about 3.5 [19,20]. Rachman et al. [31] reported that SDI fluctuated depending on factors not related to RO fouling, and Yiantsios et al. [32] suggested that *SDI* should not be used to predict RO fouling. Many analyses and studies have been conducted on the development of a new index such as the modified fouling index (MFI) [33,34], but none have been authorized and so *SDI* is still widely used.

#### 4.2.2. Membrane Biofilm Formation Rate (mBFR) as a Biofouling Index

One of the major problems in seawater desalination plants is biofouling, in which microorganisms grow, causing biofilm formation on the RO membrane surface and deterioration of the RO membrane performance. It is quite challenging to control biofouling and RO membrane replacement is performed in many cases due to the difficulty in recovering the membrane, even by chemical cleaning. There are several biofouling indices that focus on the substrate of microorganism growth, such as phosphate and dissolved organic carbon (DOC) concentration. In particular, phosphate concentration in seawater is usually lower than the other components such as carbon and nitrogen, taking the requirement ratio of microbial growth into account. Therefore, controlling the phosphate concentration is an effective method of biofouling control. However, the attachment and adherence of microorganisms onto the membrane surface may also trigger biofouling, and therefore, such indices are insufficient for biofouling.

**Figure 2:**
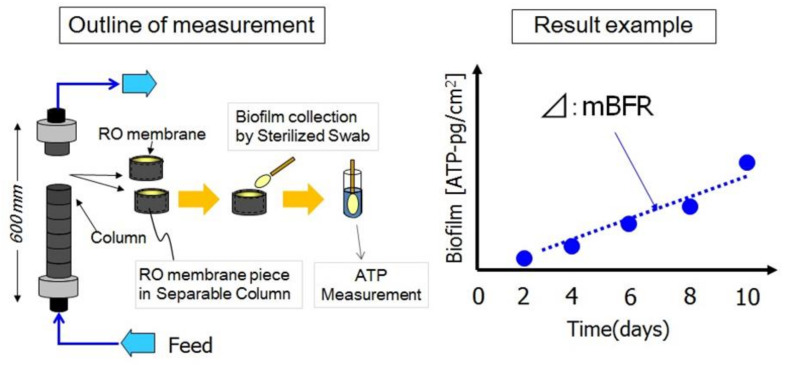
The schematic apparatus and method for the measurement of mBFR.

Therefore, the authors’ group developed the membrane biofilm formation rate (mBFR) as an index to evaluate biofouling potential [13,17,35,36,37]. mBFR is used to evaluate the biofilm formation rate on the RO membrane surface by the rate of increase of adenosine triphosphate (ATP), while sample seawater continuously flows into columns equipped with the RO membrane, as shown in Figure 2. It is an overall evaluation method for the attachment of microorganisms onto the membrane surface, as well as the microbial growth. A close correspondence between the mBFR values and the degree of biofouling has been confirmed, indicating that mBFR is a useful biofouling index [13,36,37]. For example, at Umm Al Houl SWRO Plant in Qatar, the evaluation of process water at the plant identified a point of high biofouling potential and contributed to plant stabilization [38]. Biofouling evaluation technology based on ATP measurements according to a similar concept to that of mBFR has also been reported [39].

### 4.3. Chemical Dosage of the Membrane for Sterilization and Protection from Oxidation Deterioration

The pipelines and tanks between the pre-treatment and RO process are periodically sterilized by chlorination according to a similar method as that described for the seawater intake. However, poly-amide (PA) RO membranes are not sufficiently resistant to oxidants. When a PA membrane comes into contact with oxidants, such as the residual chlorine for pipeline sterilization, the molecular structure of the RO membrane changes and its performance is irreversibly deteriorated [40]. Such an oxidation deterioration is commonly detected by the Fujiwara method [41], which is based on chromogenic detection of oxidation deterioration by chlorine contact. However, this method is not useful for a quantitative evaluation. The elemental composition on the membrane surface can be measured through electron spectroscopy for chemical analysis (ESCA), whereby the elemental ratio of oxygen and the halogen chlorine in contact with the membrane increases. Sugita et al. [42] focused on detecting bromine through ESCA in the case of a SWRO membrane used in the treatment of seawater and indicated that the Br/C ratio through ESCA quantitatively explains the RO membrane performance (permeability and boron rejection). Improved accuracy and the development of technology to detect membrane deterioration by other oxidants are expected to be established.

In order to suppress oxidation deterioration by chlorine neutralization, sodium bisulphite (SBS) is commonly added to the feedwater before the RO process. However, heavy metals in seawater generate oxidants in a catalytic reaction with SBS and it is necessary to take into account the heavy metal content of the seawater [43,44,45]. For example, in Shuqaiq Phase-II Seawater Desalination Plant (212,000 m^3^/day) in Saudi Arabia, oxidation deterioration still occurred while oxidation reduction potential (ORP) was monitored and controlled at less than 300 mV with a SBS dosage. Nada et al. [46] and Sommariva et al. [47] confirmed that it was due to sulphite auto-oxidation with the co-existence of Cu ion, and indicated that optimization of the SBS dosage amount, chelate agent (sodium salt of ethylenediaminetetraacetic acid (EDTA)) dosage and scale inhibitor dosage suppressed the reaction.

## 5. RO Process

### 5.1. Single Pass and Single Stage

#### 5.1.1. Basic Configuration

**Figure 3:**
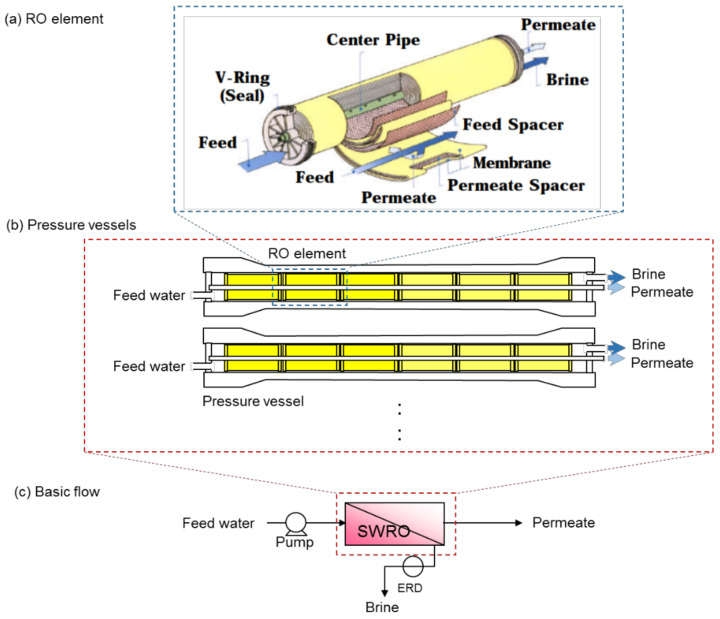
The basic configuration of the RO process. (**a**) Typical configuration of the RO element (spiral-wound type), (**b**) configuration of pressure vessels containing the RO elements, and (**c**) basic flow of single pass and single stage system.

Figure 3a shows the typical RO element configuration (spiral-wound type). The pressurized feed water is supplied to one side of the RO element, and flows in the feed-side space between RO membranes. The permeate water is collected through a center pipe, and concentrate (brine) is discharged from the other side of the RO element. RO elements are installed in a pressure vessel, usually six to eight elements in series, as shown in Figure 3b. In a seawater desalination plant, single pass and single stage is the basic configuration, and depending on its capacity, many pressure vessels are arranged in parallel [48] (see Figure 3). Herein, the brine upstream of the element is supplied as the feed water of the next element, thus the salt concentration of the feed water becomes higher as it flows downstream. This creates a tendency for high flux (due to the low osmotic pressure) and high permeate quality in the upper-stream element. Based on this tendency, the permeate of the upper-stream elements, which are obtained at the front side of the vessel, and that of downstream elements, which are obtained at the rear side of a vessel, are independently obtained and called a “partial split” [48,49]. Due to the relatively poor quality permeate at the rear side, the front and the rear permeates are occasionally used for different applications, and only the rear side permeate is occasionally additionally treated to improve the permeate water quality [50]. To level the element flux at the front and rear sides, elements with low membrane permeability are occasionally installed in the front side and elements with high membrane permeability are installed in the rear side [48,51], because high flux operation tends to cause severe fouling.

#### 5.1.2. Energy Recovery Device (ERD)

A 35–45% recovery ratio is common in the case of the single pass and single stage, which minimizes the total production cost, including the capital, operation, and energy cost of all processes, including not only the RO process but also the intake and pre-treatment [52,53]. The performance improvement and cost reduction of the RO membrane and the technical improvements to the energy recovery device (ERD) to reuse the residual energy of the RO brine contribute to reducing the production cost [54].

**Figure 4:**
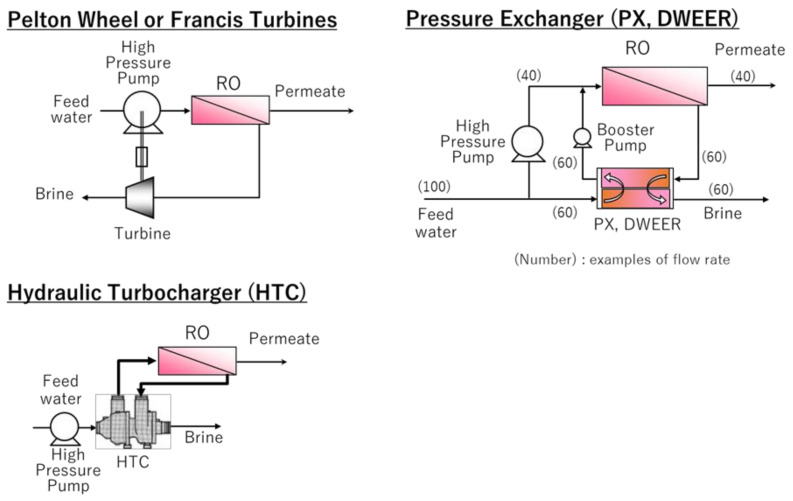
The schematic flow of the typical ERDs (Pelton wheel, Francis Turbines, Hydraulic turbocharger and Pressure exchanger (PX and DWEER).

In the 1980s, an ERD was installed to mechanically assist the high-pressure pump via a shaft with a rotating turbine, using the hydraulic energy of RO brine (see Figure 4). A typical ERD is the “Pelton wheel”, which has been adopted at several seawater desalination plants, such as Alicante in Spain, Trinidad and Tobago, Ummluji in Saudi Arabia, and Al Dur in Bahrain. Another type of ERD is the “Francis turbine”, adopted at Al-Jubail in Saudi Arabia [54,55,56]. These ERDs have drawbacks including low recovery ratios (75–85% at maximum) [54,55] and short flexibility of operation due to rapid changes of efficiency, along with the changes in the flow rate and pressure [55].

In the 1990s, a new ERD, the “Hydraulic Turbocharger (HTC)”, was adopted, which was equipped with a high-pressure pump in series to recover the hydraulic energy of RO brine via an impeller and turbine (see Figure 4). The HTC was widely accepted from users because it had many advantages such as ensuring operation flexibility (which was a disadvantage of the Pelton wheel and Francis turbine), it was applicable with Duplex fabrication to prevent corrosion, and has a high-energy recovery efficiency (90% at maximum) [55]. As HTC was available for the brine conversion system (BCS), which will be described later, it was adopted at Maspalomas II SWRO Seawater Desalination Plant in Gran Canaria, Spain [57].

Since the 2000s, ERDs using isobaric technology with higher efficiency such as “Pressure Exchanger (PX)” and “DWEER” have been adopted. These ERDs are equipped with a high-pressure pump arranged in parallel. “Isobaric” means the flow rate of the feedwater and RO brine should be equal, as shown in Figure 4. The Pelton wheel, Francis turbine, and HTC described above convert hydraulic energy to mechanical energy, then convert it back to hydraulic energy again, so there is a limit to the energy recovery efficiency. On the other hand, PX and DWEER push (pressurize) RO feedwater using the RO brine pressure. Therefore, the hydraulic energy of RO brine is directly converted to the hydraulic energy of the feedwater, which minimizes the energy loss and improves the energy efficiency to around 95% [54,55,58].

DWEER transfers RO brine pressure to feedwater via pistons in two pressure vessels. Therefore, it requires the moving range of pistons and pressure vessels with similar lengths to the RO vessels, but it is an advantage that RO feedwater and brine are physically separated and limits the contamination of feedwater and brine [55,59]. DWEER was adopted at the Tuas Desalination Plant (136,000 m^3^/day) in Singapore, for example. PX transfers RO brine pressure to feedwater in a lotus-root-shape ceramic cartridge rotating at high speed. Different from DWEER, there is a part where brine water is attached directly with feedwater without a piston, bringing slightly more contamination of brine to the feedwater, which affects the increase in operating pressure [60]. However, many large seawater desalination plants such as Perth in Australia (160,000 m^3^/day), Hamma in Algeria (200,000 m^3^/day) and Hadera in Israel (274,000 m^3^/day) adopt PX because it is simple and small-sized.

### 5.2. Multi-Pass to Improve Permeate Water Quality

**Figure 5:**
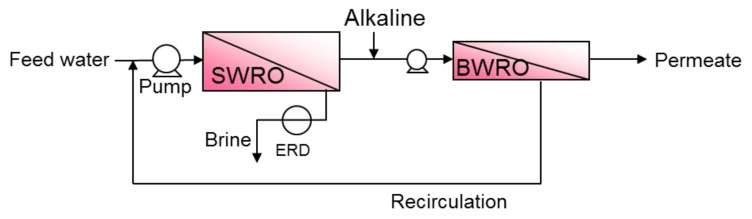
The basic flow configuration of the two-pass system. Alkaline is usually dosed into SWRO permeate at the first pass (i.e., feed water of the second pass) to enhance boron removal.

The two-pass system, where the permeate of the first pass (SWRO) is treated again with low-pressure brackish water RO (BWRO) as shown in Figure 5, is usually adopted in the case of insufficient water quality in the single pass and single stage.

**Figure 6:**
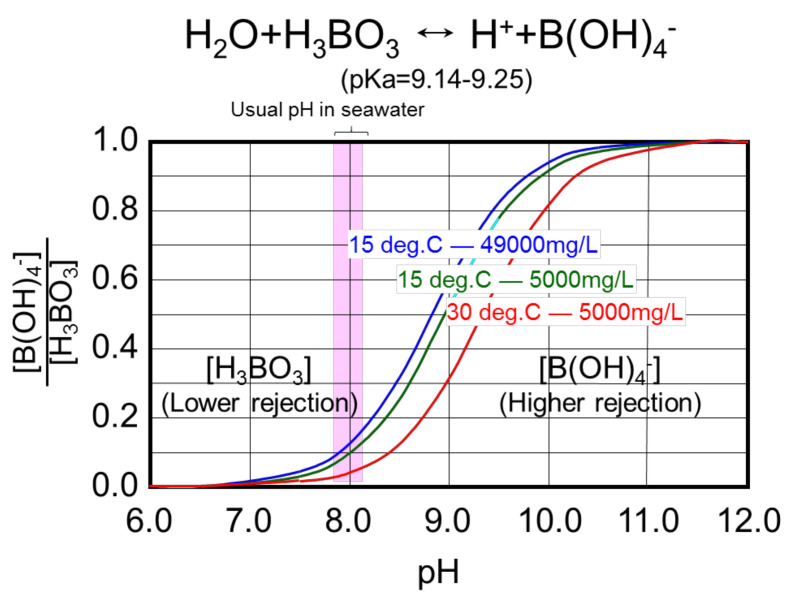
The boron dissociative equilibrium. Higher pH raises the ratio of B(OH)_4_^−^, which enhances rejection due to the electric repulsion effect of the RO membrane surface.

Boron is often focused on as a target of water quality, as is salt concentration, especially in the case of a plant that produces water for irrigation and drinking [61]. Seawater includes small amounts of boron, approximately 4.6 mg/L on average [62], which is around 20 times that of surface water, and uptake of large amounts of boron is harmful for human health and plants [61]. The WHO has established a guideline to regulate the boron concentration in drinking water. In 2011, the regulation was relaxed from 0.5 mg/L to 2.4 mg/L, but some plants still require a low boron concentration because boron has a negative effect on agricultural crops such as oranges. The boron dissociative equilibrium is shown in Figure 6. Boron predominantly exists as a form of boric acid (H_3_BO_3_) in seawater [63,64,65] because pKa is 9.14–9.25. Boric acid can be rejected easily with a RO membrane if it is dissociated but it is relatively difficult at a neutral pH without electric repulsion, because the molecular size of boric acid is small: approximately 4 angstrom.

Therefore, there are many studies on the improvement of boron removal [61] and one of the proven and popular solutions among them is that the pH is raised (alkaline is dosed) in the second pass feedwater (i.e., first pass permeate) to dissociate neutral boric acid to ions (B(OH)_4_^−^), because it has a negative charge, which enhances the rejection efficiency due to the electric repulsion effect of the membrane surface. As alkaline over-dosage increases the chemical cost and promotes membrane deterioration, it requires the optimal alkaline dosage and improvement of boron rejection at the first pass. Precise pore size control technology is important because there is a strong relation between the pore size of the RO membrane measured by positron annihilation lifetime spectroscopy (PALS) and boron rejection of the RO membrane [4,66].

### 5.3. Multi-Stage for High Recovery Ratio

**Figure 7:**
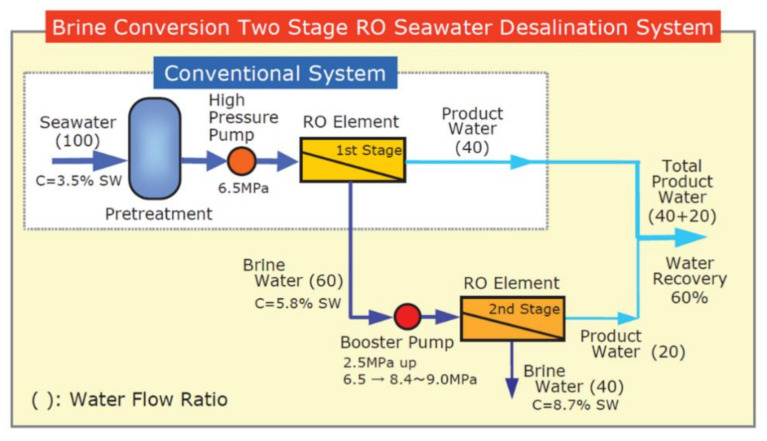
The schematic flow configuration of the brine conversion two stage RO system (BCS) with an example of flow balance [67].

In the two-stage system, the RO brine can be treated further at the single stage. The two-stage system acquires the permeate from concentrated seawater, which makes the permeate quality poorer than that of the single stage but achieves a higher recovery ratio to efficiently use the pre-treated water. In the 1990s, a Brine Conversion System (BCS), where RO brine at the first stage was boosted and fed into further RO treatment, was developed by the authors’ group [67,68,69,70]. Figure 7 shows an example of the flow. The conventional single stage recovers 40% of feedwater as permeate and generates 60% as brine. It is boosted to 10 MPa and supplied into the second-stage RO, which produces 33% of the first-stage brine (namely, 20% of feed seawater), in total, 60% of the feedwater is able to be produced as permeate in this system. BCS can easily be retrofitted into the conventional single stage, and realizes 1.5 times the product compared to the single stage [57]. To realize a BCS at seawater desalination, a membrane element with high-pressure resistance such as 10 MPa is required. Toray developed the high-pressure resistance membrane SU820BCM [5,6,7], and installed it into many large seawater desalination plants such as in Trinidad and Tobago (136,000 m^3^/day) and in the Canary Islands (14,000 m^3^/day) [68].

With the improvement of ERD performance, which meant brine energy loss was reduced and a lower recovery ratio became more feasible, and the reduced cost of pre-treatment and the demand for higher product water quality, the advantages became less in the 21st century, and installation of BCS declined. However, BCS was investigated again in the Funding Program for World-Leading Innovative R&D on Science and Technology (FIRST) program, and a new BCS was introduced, named the Low-Pressure Multi-staged System (LMS) [71,72,73,74], which could realize a 55–60% recovery ratio at low operational cost, combined with the high performance RO membrane [75] and specific energy recovery devices. LMS aims to reduce the load of the lead RO elements, which tend to cause fouling, by reducing the flux of the lead elements. A pilot test was carried out and technically demonstrated in Al-Jubail in Saudi Arabia [73]. These two-stage systems continue to be investigated [76,77].

### 5.4. Hybrid System

#### 5.4.1. Integrated System of Sewage Reclamation and Seawater Desalination

**Figure 8:**
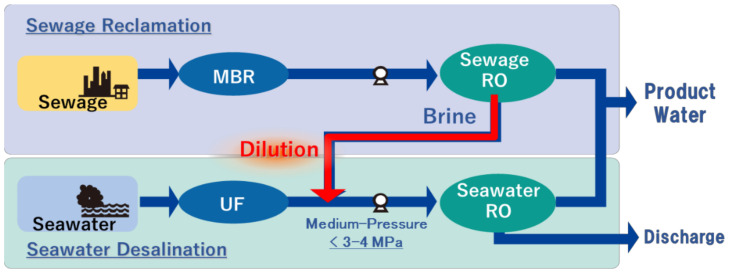
The schematic flow configuration of the integrated system of sewage reclamation and seawater desalination at “Water Plaza” in Kitakyushu, Japan. RO brine of sewage reclamation system, which is normally wasted, is reused for seawater dilution to reduce the osmotic pressure of feed water of the SWRO desalination system. It is expected that a more than 30% reduction of energy consumption is achieved in seawater desalination systems.

Today, the integrated system is being used widely in single pass and single stage, and multi-pass and multi-stage, which have been described above, and specific systems with high efficiency aiming to achieve further energy saving and cost reduction are currently being investigated. For example, in a system that integrates seawater desalination and sewage reclamation, RO brine water discharged from the sewage reclamation process dilutes seawater to reduce the osmotic pressure and reduce the energy consumption of SWRO treatment [78,79,80]. The authors’ group constructed a facility named “Water Plaza” in Kitakyushu, Japan, including a demonstration plant of 1400 m^3^/day of product water as shown in Figure 8. Influent sewage of the primary sedimentation tank is treated through a membrane bioreactor (MBR), and the effluent is supplied into BWRO and the permeate water is used as product water. The brine water of BWRO and seawater pre-treated with a UF membrane are mixed at a 50:50 ratio and supplied into the SWRO process to acquire further product water with lower applied pressure. The main technical challenge of this system was to overcome biofouling of the SWRO. The biofouling potential, measured through mBFR as described above, of the mixed water was much higher than that of UF pre-treated seawater and BWRO brine, but the optimal biocide dosage succeeded in suppressing the biofouling and stable operation was achieved. The product water was supplied to an electric power plant and used as boiler water, and it is estimated that it achieved more than 30% energy saving in seawater desalination [79,80]. According to the above mentioned result, Hitachi and New Energy and Industrial Technology Development Organization (NEDO) are currently progressing with a business demonstration at 6250 m^3^/day of product water in South Africa.

#### 5.4.2. Variable Salinity Desalination (VSD)

Variable Salinity Desalination System (VSD) was proposed and demonstrated at Marina East in Singapore, where feedwater is shifted seasonally between seawater and low concentration reservoir water [81,82]. VSD configures a two-pass system when using seawater as feedwater to obtain good quality product water. It is configured in single or two stages by changing the line connection when using low concentration water as feedwater. VSD is installed to minimize energy consumption to achieve water production and product quality according to the local water situation.

#### 5.4.3. Closed Circuit Desalination System (CCD)

**Figure 9:**
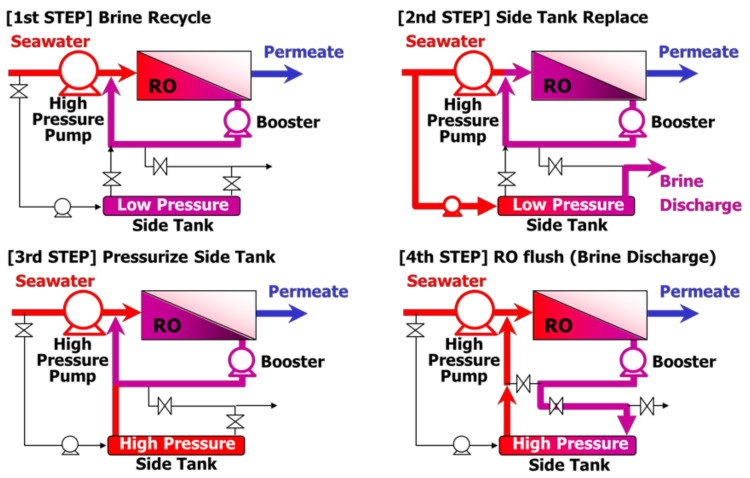
The sequential operation of CCD. At the first step, RO treatment is carried out with brine recirculation to feed water. The concentration of RO feed water gradually increases, then the brine stored in the side tank is discharged by pushing new seawater (second step). Then, the side tank is connected with the recirculation line to increase the pressure in the side tank (third step). Finally, the line is switched to push the seawater stored in the side tank to use feed water by replacing the condensed brine in the side tank (4th step), going back to the first step.

Closed Circuit Desalination System (CCD) is a technology developed by Desalitech in Israel. Shifting the operation mode of CCS sequentially, as shown in Figure 9, realizes energy saving with a relatively small amount of RO elements without ERD [83,84,85,86,87,88]. In CCD, operation starts at low pressure with recirculation of RO brine to feedwater, and then gradually increases the pressure as well as filling the pressure-resistant side tank with seawater. When the salt concentration of the RO feedwater becomes high, the seawater in the side tank is pushed by RO brine with high pressure and sent as RO feedwater. CCD is a well-thought-out system. Desalitech verified CCD in the Mediterranean Sea (TDS: 41,400 mg/L) with an energy consumption of 1.65–1.85 kWh/m^3^ [83]. Less than 1.5 kWh/m^3^ while optimizing the flux and recovery ratio was also reported [88]. CCD has the advantage of being able to increase the recovery ratio with fewer RO elements in series [88], and Stover proposed that CCD is useful in the water treatment of oil and gas with a high recovery ratio [87]. On the other hand, it has been reported that a conventional system such as single stage and multi-stage is better in the thermodynamic theoretical calculation than CCD [88]. Although CCD has several unique features and merits as described above, CCD is still difficult to apply because the load on the RO membrane is very high due to exposure to rapid and frequent condition changes, and the valve system is complicated and adequate control is not easy when the surrounding conditions such as feedwater quality and temperature are changing.

#### 5.4.4. Deep Sea RO

An RO desalination system submerged in the deep sea (“Deep-sea RO”) has also been investigated [89,90]. A deep-sea RO system is operated under the sea with a low recovery ratio, which has several expectable advantages: (1) it reduces the pressure applied on the RO membrane, (2) only permeate is required to pump up to the land (i.e., pumping cost is less than half), and (3) pre-treatment is expected to be less due to the feedwater being obtained from deep seawater as mentioned above. One of the developers, DXW Water Technologies, reported that the energy consumption of deep-sea RO is approximately 1.2–1.3 kWh/m^3^ [90,91]. However, this system requires heavy equipment for the pipeline and electricity, and system maintenance is difficult. There is a potential to further develop this system but there are obstacles to overcome to commercialize deep-sea RO on a large scale.

As described above, various unique systems have been developed to achieve energy saving and cost reduction. Some are expected to overcome the technical hurdles and be commercialized for a wide application.

### 5.5. Tools for Basic Design of a Seawater Desalination Plant

#### 5.5.1. Calculation Software for the Basic Design of Seawater Desalination Plants

For the design of RO processes such as those described above, engineers should evaluate the plant performance by calculating whether the product water quality meets the demand values, how much pressure is required for the feedwater high-pressure pump, etc. The calculation of plant performance includes the RO permeation for all elements, taking the plants configuration and circumstances into account, such as the feedwater quality composition, water temperature, type and location of RO elements, configuration of vessel/stage/pass, and recirculation flow. RO permeation should be simulated and calculated strictly based on the theory of concentration polarization on the RO membrane surface, taking each RO element’s features into account [69,92,93,94,95], such as the net spacer of the feedwater, membrane performance dependency of pressure, temperature, salt concentration, and pH. Simulation software for these calculations is provided by each RO membrane manufacturer and it is recommended to undergo a trial utilization. For example, “TorayDS2” is one of the design softwares provided by Toray, which is able to be downloaded freely after simple registration.

The knowledge of the fundamental principle of concentration polarization theory is helpful to understand RO plant performance and the basic design. The basic equations of concentration polarization consists of the following equations (these equations were simplified (reflection coefficient is assumed to be zero) for easy understanding);

(2) $$J_{V}=L_{P}\cdot ∆P$$

(3) $$J_{S}=P\left(C_{M}-C_{P}\right)$$

(4) $$\frac{C_{M}-C_{P}}{C_{B}-C_{P}}=\exp\left(\frac{J_{V}}{k}\right),\;k=\frac{D}{\delta }$$

(5) $$C_{P}=\frac{J_{S}}{J_{V}}$$

where *J_V_* is volume flux (m^3^/m^2^/s), *L_P_* is solution permeability (m^3^/m^2^/Pa/s), Δ*P* is effective pressure (Pa), *J_S_* is salt flux (kg/m^2^/s), *P* is salt permeability (m/s), *C_M_* is salt concentration at feed side of membrane surface (kg/m^3^), *C_B_* is salt concentration in the bulk (kg/m^3^), *C_P_* is salt concentration of permeate (kg/m^3^), *k* is mass transfer coefficient (m/s), *D* is diffusivity (m^2^/s), *δ* is thickness of boundary layer (m). The detail of this theory is referred to in other papers such as [69,92,93,94,95], but most importantly, the driving force of water (solution) permeation and salt permeation are effective pressure (i.e., (added pressure)—(osmotic pressure between feed and permeate side)) and salt concentration differs between feed and permeate side, respectively. In addition, salt concentration of permeate is determined by the ratio of *J_S_* and *J_V_* as shown in Equation (5). Thus, higher flux tends to induce better permeate water quality. However, higher flux also raises *C_M_* (Equation (4)), which also causes a decrease in *J_V_* and an increase in *J_S_*. Therefore, the effect of water quality improvement of higher flux is limited. In these equations, *L_P_*, *P* and *k* are the values to express the membrane and element performance, but these values are subject to change depending on the circumstances. Therefore, the total calculation is quite complicated. As described above, the usage of the calculation software is strongly recommended.

#### 5.5.2. Determination of Scale Inhibitor Dosage

In the RO process, feedwater becomes concentrated gradually through RO treatment. Therefore, scaling on the membrane surface due to the condensation of the feedwater component over the solubility might occur, depending on the feedwater quality and recovery ratio (condensation ratio). Scaling deteriorates the permeability and salt rejection due to scale attaching on the membrane surface. Moreover, sharp salt crystals scratch the membrane surface, causing irreversible fouling [95]. To avoid scaling, a scale inhibiter is dosed. Polyphosphonate- and polyacrylate-based scale inhibitors are widely used in desalination plants, and a suitable type and dosage amount depends on the plant circumstances and the scaling potential of the feedwater. To determine the dosage, the effects of scale inhibitors should be estimated quantitatively while taking into consideration the water temperature, condensation degree on the membrane surface, and salt solubility with ion strength impact. This calculation is quite complicated and the scale inhibitor suppliers recommend using a simulation tool. The correct amount of scale inhibitor should be used because over dosage may cause RO biofouling [38,96] and increases operating costs.

## 6. Concluding Remarks

In the 21st century, RO membrane desalination technologies are indispensable for modern life. This paper describes the general issues and history of the technologies for the basic design, operation and maintenance of seawater desalination plants. The technologies for seawater desalination plants are still evolving and there are various options and combinations to meet new challenges. We hope this paper is helpful for engineers engaged in operations and process design of seawater desalination plants.

## Figures and Tables

**Table 1:** The timeline of the basic history of SWRO membranes.

Year	Events
1950s	Start of exploratory research on RO membranes at Univ. of Florida followed by ULCA.
1960	Initial type of CA asymmetric membrane was developed.
Middle 1960s	General Atomics started the commercialization of CA flat sheet membrane (spiral-wound type).
1968	DuPont launched the linear PA hollow fibre membrane. Toray started R&D on spiral wound type of CA and PA flat sheet membrane.
1972	Toyobo started R&D on RO membrane.
1973	Nitto Denko started R&D on RO membrane.
1977	Cadotte and his colleagues succeeded in developing a cross-linked aromatic PA membrane.
1980s	Toray developed a cross-linked polyether composite membrane followed by ultra-thin cross-linked PA composite membrane.
1985	NittoDenko acquired Hydranautics.
1987	Dow acquired FilmTech.
Current	PA composite membranes are main players of SWRO membrane, and main four companies (Toray, DuPont (formerly Dow), Hydranautics and LG) share more than 90% market in the world.

**Table 2:** The typical pre-treatment types and their basic pre-treatment process flow configurations. Their main advantages and disadvantages are also summarized with example desalination plants that have adopted these pre-treatment processes (plant information was summarized with referring yearbooks published from IDA [21,22,23,24,25,26,27] and the parenthesized values indicate the product water capacity of each plant (×10^3^ m^3^/day)).

Pre-Treatment Type /Basic Process Flow	Advantages/Disadvantages	Plant Examples
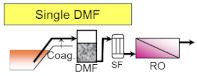	- Conventional process applied at many plants - Proven technology and easy operation - Unstable efficiency along with the quality of seawater	Sydney (250) Al Jubail (90) Hamma (200) Perth (130) Alicante II (65) Carboneras (120) Ashkelon (330) Tenes (200)
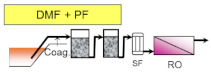	- Better water quality than single DMF - Unstable efficiency along with the quality of seawater	Okinawa (40) Mostaganem (200) Torrevieja (240) Guadalentin (210)
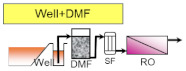	- Better and stable water quality - Limited applicable location - Limited life of well and necessity for renewal	Fukuoka (50) Alicante I (64)
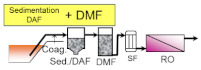	- Better and stable water quality - Applicable even for red tide and oil pollution - Large consumption of chemicals-High operation cost	Tuas (130) Point Lisas (130)
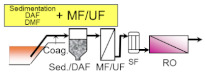	- Excellent and stable water quality - Stable operation against fluctuation of seawater quality - High initial and operation cost	Shuweikh (130) London (150) Tuas II (380) Tianjin (100)
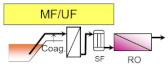	- Excellent and stable water quality - Compact footprint - CDifficulty in corresponding to oils	Magtaa (500) Adelaide (270) Perth II (150) Carlsbad (190)

## References

[1] Fujiwara M., Aoshima Y., Miki T. (2012). Development of Polyamide Composite Reverse Osmosis Membrane and Reverse Osmosis, Membrane System: A case study of Toray Industries, Inc..

[2] Global Water Intelligence DesalData, Desalination Projects, December 2020. https://www.desaldata.com/projects.

[3] Kurihara M., Takeuchi H. (2018). SWRO-PRO system in “Mega-ton Water System” for energy reduction and low environmental impact. Water.

[4] Kurihara M., Sasaki T. (2017). The most advanced membrane analysis and the save-energy type membrane-low-pressure seawater reverse osmosis membrane developed by “Mega-ton Water System” project. Compr. Membr. Eng. II.

[5] Kurihara M., Hanakawa M. (2013). Mega-ton Water System: Japanese national research and development project on seawater desalination and wastewater reclamation. Desalination.

[6] Cadotte J.E. (1977). Reverse Osmosis Membrane. U.S. Patent.

[7] Kurihara M., Fusaoka Y., Uemura T. Development of Excellent Performance Crosslinked Aromatic Polyamide Composite Membranes and their Practical Application for seawater desalination. Proceedings of the International Congress on Membranes and Membrane Processes.

[8] Kurihara M., Himeshima Y., Uemura T. Crosslinked aromatic polyamide ultra-thin composite membrane from 1,3,5-triaminobenzene. Proceedings of the International Congress on Membranes and Membrane Processes.

[9] Kurihara M., Fusaoka Y., Sasaki T., Bairinji R., Uemura T. (1994). Development of crosslinked fully aromatic polyamide ultra-thin composite membranes for seawater desalination. Desalination.

[10] Global Water Intelligence (2018). DesalData, September 2018.

[11] Wilf M. (2007). The Guidebook to Membrane Desalination Technology.

[12] Messimer T.M., Maliva R.G. (2018). Environmental issues in seawater reverse osmosis desalination: Intakes and outfalls. Desalination.

[13] Ito Y., Hanada S., Kitade T., Tanaka Y., Kurihara M. Clarification of impact of biofouling triggered by chemical Addition for Designing of Mega-Ton SWRO Plant. Proceedings of the IDA World Congress.

[14] Nigeme E., Yoshihara S. (2004). Research on low cost pumping of deep seawater and its regional business promotion. Deep Ocean Water Res..

[15] Shahabi M.P., McHugh A., Ho G. (2015). Environmental and economic assessment of beach well intake versus open intake for seawater reverse osmosis desalination. Desalination.

[16] Missimer T.M., Ghaffour N., Dehwah A.H.A., Rachman R., Maliva R.G., Amy G. (2013). Subsurface intakes for seawater reverse osmosis facilities: Capacity limitation, water quality improvement, and economics. Desalination.

[17] Kurihara M., Takeuchi H., Ito Y. (2018). Reliable seawater desalination system based on the membrane technology and the biotechnology considering Environmental Impacts. Environments.

[18] Kiernan J. Construction and Startup of the Largest Seawater Desalination Plant in the Western Hemisphere. Proceedings of the IDA World Congress.

[19] Sallangos O.V., Kantilaftis E. (2001). Operating experience of the Dhekelia seawater desalination plant. Desalination.

[20] Sallagos O.V. The Importance of Pretreatment in Reverse Osmosis Desalination. Proceedings of the IDA RO Pretreatment Workshops.

[21] IDA (2009). IDA Water Security Yearbook 2008–2009.

[22] IDA (2010). IDA Water Security Yearbook 2009–2010.

[23] IDA (2014). IDA Desalination Yearbook 2013–2014.

[24] IDA (2015). IDA Desalination Yearbook 2014–2015.

[25] IDA (2017). IDA Desalination Yearbook 2016–2017.

[26] IDA (2018). IDA Desalination Yearbook 2017–2018.

[27] IDA (2020). IDA Water Security Handbook 2019–2020.

[28] Sim L.N., Chong T.H., Taheri A.H., Sim S.T.V., Lai L., Kranz W.B., Fane A.G. (2018). A review of fouling indices and monitoring techniques for reverse osmosis. Desalination.

[29] ASTM (1995). ASTM D4189-95, Standard Test Method for Silt Density Index (SDI) of Water.

[30] ASTM (2007). ASTM D4189-07, Standard Test Method for Silt Density Index (SDI) of Water.

[31] Rachman R.M., Ghaffour N., Wali F., Amy G.L. (2013). Assessment of silt density index (SDI) as fouling propensity parameter in reverse osmosis (RO) desalination systems. Desalin. Water Treat..

[32] Yiantsios S.G., Karabelas A.J. (2002). An assessment of the Silt Density Index based on RO membrane colloidal fouling experiments with iron oxide particles. Desalination.

[33] Rabie H.R., Cote P., Adams N. (2001). A method for assessing membrane fouling in pilot and full-scale systems. Desalination.

[34] Schippers J.C. (2013). SDI and MFI workshop: Conclusions and recommendations. Desalin. Water Treat..

[35] Ito Y., Kantani S., Uemura T., Kitade T. (2007). Method for Operating Reverse Osmosis Membrane Filtration Plant, and Reverse Osmosis Membrane Filtration Plant. EU Patent.

[36] Ito Y., Kantani S., Maeda T., Okubo K., Taniguchi M. Innovative Biofouling Monitoring Device and Its Criteria for Reverse Osmosis Plant Operation and Optimization. Proceedings of the IDA World Congress.

[37] Kurihara M., Ito Y. (2020). Sustainable seawater reverse osmosis desalination as green desalination in the 21st century. J. Membr. Sci. Res..

[38] Cortes P., Hijos G. Performance Study of Sulphuric Acid Shocks in Reverse Osmosis Membranes in the Umm Al Houl Project SWRO Plant. Proceedings of the IDA World Congress.

[39] Abushaban A., Salinas-Rodriguez S.G., Kapala M., Pastorelli D., Schippers J.C., Mondal S., Gaueli S., Kennedy M.D. (2020). Monitoring biofouling potential using ATP-based bacterial growth potential in SWRO pre-treatment of a full-scale plant. Membranes.

[40] Uemura T., Kurihara M. (2003). Chlorine resistance of reverse osmosis membranes and changes in membrane structure and performance caused by chlorination degradation. Bull. Soc. Sea Water Sci. Jpn..

[41] Uno T., Okumura K., Kuroda Y. (1981). Mechanism of Fujiwara reaction: Structural investigation of reaction products from benzotrichloride. J. Org. Chem..

[42] Sugita K., Fusaoka Y., Kotera K., Fujiwara K., Uemura T. Quantitative Analysis on the Oxidation of SWRO Membrane. Proceedings of the IDA World Congress.

[43] Brandt C., van Eldik R. (1995). Transition metal-catalyzed oxidation of sulphur(IV) oxides atmospheric-relevant processes and mechanisms. Chem. Rev..

[44] Neta P., Huie R.E. (1985). Free-radical chemistry of sulphite. Environ. Health Perspect..

[45] Kuo D.T.E., Kirk D.W., Jia C.Q. (2006). The chemistry of aqueous S(IV)-Fe-O2 system: State of the art. J. Sulfur Chem..

[46] Nada N., Attenborough T., Ito Y., Maeda Y., Tokunaga K., Iwahashi H. (2011). SWRO drinking water project in Shuqaiq: Advanced BWRO, membrane oxidation, and scaling. IDA J..

[47] Sommariva C., Attenborough A., Poggi F., Al-Mahdi A.A.M., Hori T., Tokunaga T.K., Ito Y., Maeda Y. (2012). Matching hollow-fiber with spiral-wound membranes: Process compatibility and optimization. IDA J..

[48] Voutchkov N. (2011). Desalination Cost Assessment and Management.

[49] Mubeen F.M. Unique Design Features of Commercial SWRO Plants. Proceedings of the IDA World Congress.

[50] Alnouri S.Y., Linke P. Optimal SWRO Network Synthesis with Specifications for Multiple Permeate Components. Proceedings of the IDA World Congress.

[51] Yamamura H., Fusaoka Y., Kurihara M. (2002). Reverse Osmosis Separation Unit and Operation Method. J.P. Patent.

[52] Bhattacharyya D., Ridgway H., Wilf M. Future Research Needs to Move Technology forward by Magnitude Note Increment. Proceedings of the Pacific Rim “Quantum Leap” Membrane Search Symposium.

[53] Du Y., Xie L., Wang Y., Xu Y., Wang S. Optimal Design and Operation of Reverse Osmosis Desalination Process with Spiral Wound Modules. Proceedings of the IDA World Congress.

[54] Gude V.G. (2011). Energy consumption and recovery in reverse osmosis. Desalin. Water Treat..

[55] Guirguis M.J. (2011). Energy Recovery Devices in Seawater Reverse Osmosis Desalination Plants with Emphasis on Efficiency and Economical Analysis of Isobaric Versus Centrifugal Devices. Ph.D. Thesis.

[56] Faroque A. (2008). Parametric analysis of energy consumption and losses in SWCC SWRO plants utilizing energy recovery devices. Desalination.

[57] Oklejas E., Moch I., Nielsen K. (1995). Low cost incremental seawater plant capacity increase by coupling advanced pumping and RO technologies. Desalination.

[58] Penate B., Garcia-Rodriguez L. (2011). Energy optimization of existing SWRO (seawater reverse osmosis) plants with ERT (energy recovery turbines): Technical and thermoeconomic assessment. Energy.

[59] Schneider B. (2005). Selection, operation and control of a work exchanger energy recovery system based on the Singapore project. Desalination.

[60] (2008). SWRO desalination plant uses ERI pressure exchanger. Membr. Technol. September.

[61] Tu K.L., Nghiem L.D., Chivas A.R. (2010). Boron removal by reverse osmosis membranes in seawater desalination applications. Separat. Purif. Technol..

[62] Argust P. (1998). Distribution of boron in the environment. Biol. Trace Elem. Res..

[63] Busch M., Mickois W.E., Jons S., Redondo J., Write J.D. Boron Removal in Sea Water Desalination. Proceedings of the IDA World Congress.

[64] Rodriguez M., Ruiz A.F., Chilin M.F., Rico D.P. (2001). Influence of pH in the elimination of boron by means of reverse osmosis. Desalination.

[65] Hyung H., Kim J.H. (2006). A mechanistic study on boron rejection by sea water reverse osmosis membranes. J. Membr. Sci..

[66] Henmi M., Fusaoka Y., Tomioka H., Kurihara M. (2010). High performance RO membranes for desalination and wastewater reclamation and their operation results. Water Sci. Technol..

[67] Kurihara M., Yamamura H., Nakanishi T. (1999). High recovery/high pressure membranes for brine conversion SWRO process development and its performance data. Desalination.

[68] Kurihara M., Yamamura H., Nakanishi T., Jinno S. (2001). Operation and reliability of very high-recovery seawater desalination technologies by brine conversion two-stage RO desalination system. Desalination.

[69] Taniguchi M., Kurihara M., Kimura S. (2001). Behavior of a reverse osmosis plant adopting a brine conversion two-stage process and its computer simulation. J. Membr. Sci..

[70] Yamamura H., Kurihara M., Maeda K. (2001). Apparatus and Method for Multistage Reverse Osmosis Separation. U.S. Patent.

[71] Kitamura K., Yoshikawa S. Low Fouling and Energy Save Seawater Reverse Osmosis Desalination System for High Recovery Rate. Proceedings of the IDA World Congress on Desalination and Water Reuse.

[72] Kitamura K., Miyakawa H. Verification of advanced designed seawater RO system for low energy and operation cost. Proceedings of the IDA World Congress on Desalination and Water Reuse.

[73] Ayumantakath M.F., Al Shaiae M.M., Green T.N., Miyakawa H., Ito Y., Kurokawa H., Fusaoka Y., Al Amoudi A.S. Reliable Sea Water RO Operation with High Water Recovery and No-Chlorine/No-SBS Dosing in Arabian Gulf, Saudi Arabia. Proceedings of the IDA World Congress on Desalination and Water Reuse.

[74] Kishizawa N., Tsuzuki K., Hayatsu M. (2015). Low pressure multi-stage RO system developed in “Mega-ton Water System” for large-scaled SWRO plant. Desalination.

[75] Kurihara M., Sasaki T., Nakatsuji K., Kimura M., Henmi M. (2015). Low pressure SWRO membrane for desalination in the Mega-ton Water System. Desalination.

[76] Wei Q., McGovern R., Lienhard J.H.V. (2017). Saving energy with an optimized two-stage reverse osmosis system. Environ. Sci. Water Res. Technol..

[77] Wei Q., McGovern R., Lienhard J.H.V. Two-Stage Reverse Osmosis: Optimal Element Configuration and Energy Savings. Proceedings of the IDA World Congress.

[78] Sekine Y., Takabatake H., Noto K. Start of Product Water Supply from the Integrated System of Seawater Desalination and Sewage Reuse (Water Plaza). Proceedings of Water Environment Symposium.

[79] Takabatake H., Noto K., Uemura T., Ueda S. (2013). More than 30% energy saving seawater desalination system by combining with sewage reclamation. Desalin. Water Treat..

[80] Cheon J., Sekine Y., Takabatake H., Noto K., Uemura T., Ueda S. (2012). Demonstration of more than 30% of energy saving by the seawater desalination system combined with wastewater treatment system. Water Pract. Technol..

[81] Chin J.S.S., Pang C.M., Ong K.W., Seah H. Increasing Water Resources through Desalination in Singapore: Planning for a Sustainable Future. Proceedings of the IDA World Congress.

[82] Seah H., Khoo K.L., Chua S.C., Chua J.Y., Toh C.W. Cost Effective Way to Harvest Estuarine Water Variable Salinity Desalination Concept. Proceedings of the SIWW 2008.

[83] (2010). Closed Circuit RO Eliminates ERD.

[84] Stover R. High Recovery Using Closed Circuit Desalination Process for Inland Brackish and Oil and Gas Applications. Proceedings of the IDA World Congress.

[85] Efaty A. (2009). Apparatus for Continuous Closed Circuit Desalination under Variable Pressure with a Single Container. U.S. Patent.

[86] Efaty A. (2010). Continuous Closed-Circuit Desalination Apparatus without Containers. U.S. Patent.

[87] Stover R.L., Efraty N. Record Low Energy Consumption with Closed Circuit Desalination. Proceedings of the IDA World Congress.

[88] Lin S., Eimelech M. (2015). Staged reverse osmosis operation: Configurations, energy efficiency, and application potential. Desalination.

[89] Pacenti P., de Gerloni M., Reali M., Chiaramonti D., Gartner S.O., Helm P., Stohr M. (1999). Submarine seawater reverse osmosis desalination system. Desalination.

[90] (2008). A New Approach to Deep Sea RO.

[91] Vuong D.X. (2008). Depth Exposed Membrane for Water Extraction. U.S. Patent.

[92] Qiu T.Y., Davies P.A. (2015). Concentration polarization model of spiral-wound membrane modules with application to batch-mode RO desalination of brackish water. Desalination.

[93] Brian P.L.T. (1965). Concentration polarization in reverse osmosis desalination with variable flux and incomplete salt rejection. Ind. Eng. Chem. Fundam..

[94] Taniguchi M., Kihara M., Yamamura H., Kurihara M. (2002). Studies of simulation method based on concentration polarization theory for reverse osmosis seawater desalination plant. Bull. Soc. Sea Water Sci. Jpn..

[95] Matin A., Rahman F., Shafi H.Z., Zubair S.M. (2019). Scaling of reverse osmosis membranes used in water desalination: Phenomena, impact, and control; future directions. Desalination.

[96] Sweity A., Oren Y., Ronen Z., Herberg M. (2013). The influence of antiscalants on biofouling of RO membranes in seawater desalination. Wat. Res..

